# Male sex is strongly associated with IgE-sensitization to airborne but not food allergens: results up to age 24 years from the BAMSE birth cohort

**DOI:** 10.1186/s13601-020-00319-w

**Published:** 2020-05-25

**Authors:** Erik Melén, Anna Bergström, Inger Kull, Catarina Almqvist, Niklas Andersson, Anna Asarnoj, Magnus P. Borres, Antonis Georgellis, Göran Pershagen, Marit Westman, Marianne van Hage, Natalia Ballardini

**Affiliations:** 1 Department of Clinical Science and Education Södersjukhuset, Karolinska Institutet, 11883 Stockholm, Sweden; 2 Institute of Environmental Medicine, Karolinska Institutet, 17177 Stockholm, Sweden; 3 Sachs’ Children and Youth Hospital, Södersjukhuset, 11883 Stockholm, Sweden; 4 Centre for Occupational and Environmental Medicine, Region Stockholm, 113 65 Stockholm, Sweden; 5 Pediatric Allergy and Pulmonology Unit at Astrid Lindgren Children’s Hospital, Karolinska University Hospital, 171 76 Stockholm, Sweden; 6 Department of Medical Epidemiology and Biostatistics, Karolinska Institutet, 17 77 Stockholm, Sweden; 7 Department of Medicine Solna, Division of Immunology and Allergy, Karolinska Institutet and Karolinska University Hospital, Stockholm, Sweden; 8 Department of Women’s and Children’s Health, Karolinska Institutet, Stockholm, Sweden; 9 Department of Women’s and Children’s Health, Uppsala University and Thermo Fisher Scientific, Uppsala, Sweden

**Keywords:** Allergen, BAMSE, Birth cohort, Immunoglobulin E, Prevalence, Sensitization

## Abstract

**Background:**

Up to half of the population in high-income countries has allergen-specific IgE antibodies. However, data regarding sex differences of IgE-sensitization from childhood to adulthood is limited.

**Objective:**

To explore IgE-sensitization to common foods and airborne allergens in relation to sex over time in a population-based cohort followed up to young adulthood.

**Methods:**

The Swedish population-based birth cohort BAMSE includes 4089 subjects who have been followed regularly with questionnaires and clinical investigations. A recent 24-year follow-up included 3069 participants (75%). Sera collected at 4, 8, 16 and 24 years were analyzed for IgE-antibodies to 14 common foods and airborne allergens.

**Results:**

At 24 years sensitization to foods had decreased compared to previous follow-ups affecting 8.4%, while sensitization to airborne allergens was more common, affecting 42.2%. Male sex was associated with IgE-sensitization to airborne allergens at all ages (overall OR: 1.68, 95% CI 1.46–1.94) while there was no statistically significant association between sex and sensitization to food allergens (overall OR: 1.10, 95% CI 0.93–1.32). Levels of allergen-specific IgE did not differ significantly between males and females for any of the tested foods or airborne allergens at any age, following adjustment for multiple comparisons.

**Conclusion:**

IgE-sensitization to airborne allergens increases with age up to young adulthood, whereas sensitization to food allergens seems to level off. Male sex is strongly associated with IgE-sensitization to airborne allergens from early childhood up to young adulthood. In contrast, there is little evidence for associations between sex and IgE-sensitization to foods.

## Introduction

The increased prevalence of allergic diseases is a major public health concern worldwide [1]. Production of specific immunoglobin E antibodies (sIgE) against allergens constitutes a hallmark of allergic disease [2, 3]. Up to half of the adult population has sIgE [4–6], but knowledge regarding development of IgE-sensitization over time is limited. Investigation of the natural course and development of IgE-sensitization requires large longitudinal population-based studies including repeated measurements of sIgE. Such studies are few [7–10] and often do not go beyond childhood [11–16] or include only adults [17, 18].

Females and males have been shown to differ regarding sIgE-sensitization. Most studies report prevalences of IgE-sensitization that are higher in males than females at least up to adolescence [6, 7, 19–22]. The prevalence of allergic diseases also differs between females and males, and can be higher in either females or males depending on age and disease, which cannot be explained solely by differences in IgE-sensitization [23].

Exploring sex differences and patterns of IgE-sensitization over time is important since IgE-sensitization is a significant risk factor for the development of allergic disease. We therefore analyzed IgE-sensitization to foods and airborne allergens among females and males in the population-based birth cohort BAMSE [Barn/Children, Allergy, Milieu, Stockholm, Epidemiology] followed up to young adulthood (24 years of age). Our specific aim was to report IgE-sensitization at the recent follow-up at age 24 years and to evaluate possible differences between females and males regarding prevalences and levels of IgE-sensitization at ages 4, 8, 16 and 24 years.

## Methods

### Study design and study subjects

BAMSE is a population-based cohort from Stockholm, Sweden, in which newborn children were recruited from 1994 to 1996 and followed up to 24 years of age [24]. The BAMSE cohort comprised 4089 infants, corresponding to 75% of the eligible subjects [25]. When the children were 2 months (baseline) parents completed questionnaires on background factors and follow-up questionnaires including questions related to symptoms of eczema, asthma, and rhinitis were sent out and answered at 1, 2, 4, 8, 12,16 and 24 years of age.

**Fig. 1:**
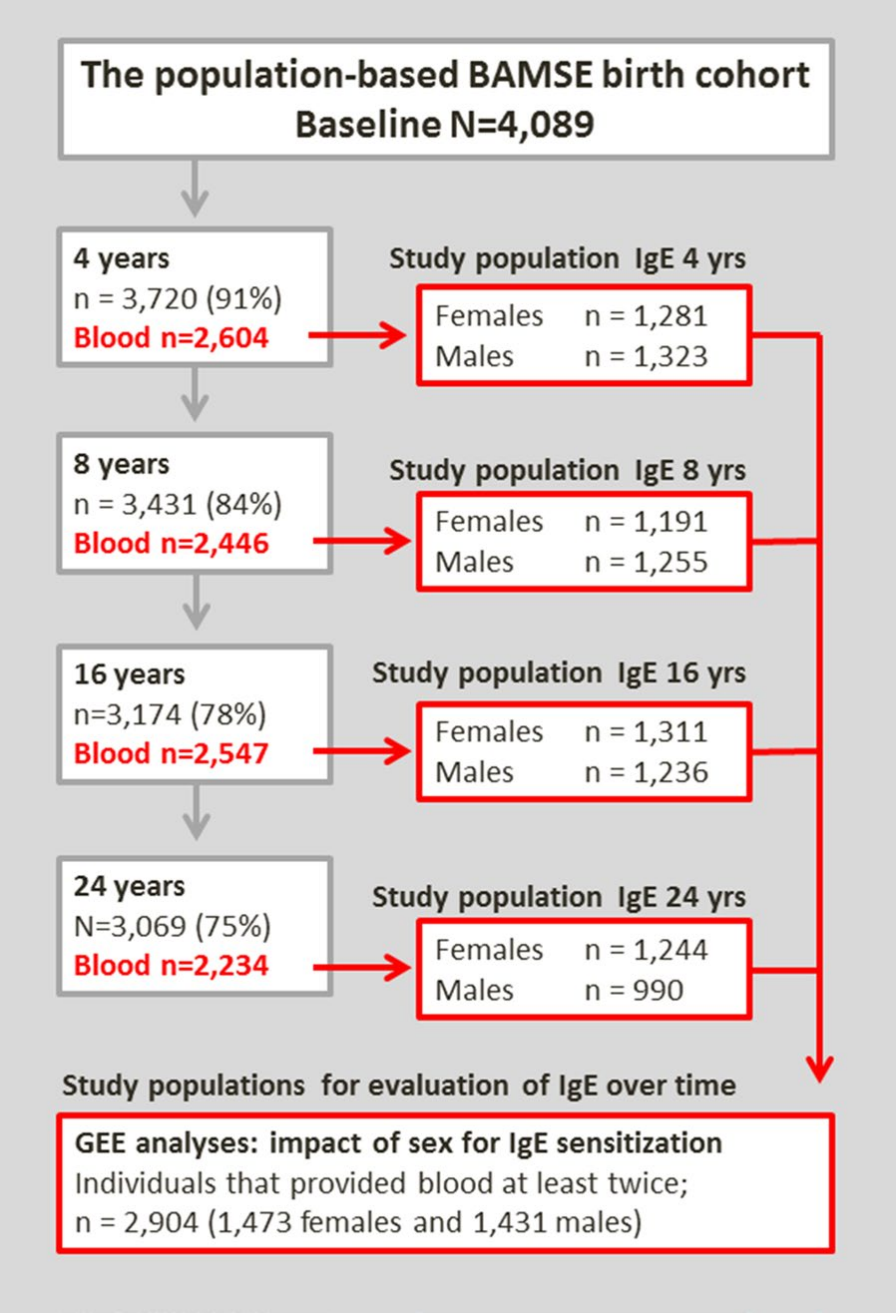
Flow chart of the current study and study-populations used for included analyses

The study populations used are shown in Fig. 1. For evaluation of IgE-sensitization at the 24-year follow-up, individuals with complete data regarding IgE-sensitization at age 24 years (n = 2234) were included. For evaluation of differences between females and males regarding prevalence of IgE-sensitization to specific allergens at ages 4, 8 and 16 years, individuals with complete data on sIgE-sensitization at the respective ages were included. For longitudinal analyses evaluating the association between sex and IgE-sensitization to foods and airborne allergens over time by use of generalized estimating equations, individuals that provided blood at least twice (n = 2904) were included.

## Definitions

### Sensitization

Allergen-specific IgE antibodies were analyzed with the ImmunoCAP System (Thermo Fisher Scientific, Uppsala, Sweden) as recommended by the manufacturer. The results were expressed in kilounits of allergen per liter, and a positive test result was defined as ≥ 0.35 kU_A_/l. Sera were analyzed for a mix of common food allergens with fx5^®^ and a mix of common airborne allergens with Phadiatop^®^ [26]. Sera that scored positive (IgE ≥ 0.35 kU_A_/l) for a mix were further analyzed for the single allergens included in the food mix (fx5; peanut, soy, wheat, milk, egg and cod) and/or the mix of airborne allergens (Phadiatop; timothy, birch, cat, dog, house dust mites (*Dermatophagoides pteronyssinus* at ages 4 years and 8 years and *Dermatophagoides pteronyssinus* and/or *Dermatophagoides farinae* at ages 16 years and 24 years), mugwort, horse, and *Cladosporium herbarum*). All samples were analyzed at the Department of Clinical Immunology Karolinska University Hospital Solna, Stockholm, Sweden.

*IgE*-*sensitization to foods* was defined as sIgE ≥ 0.35 kU_A_/l to one or more of the tested food allergens and *sensitization to airborne allergens* was defined as sIgE ≥ 0.35 kU_A_/l to one or more of the tested airborne allergens. *Any IgE*-*sensitization* was defined as sIgE ≥ 0.35 kU_A_/l to one or more of the tested food and/or airborne allergens (14 in total).

### Statistical analysis

Chi square tests were used for evaluation of differences between females and males for dichotomous outcomes. When exploring differences in IgE-sensitization to the 14 included allergens between females and males we used Bonferroni correction and a *p* value lower than 0.004 (0.05/14) was considered statistically significant. The median level of IgE was calculated by adding levels for all positive (≥ 0.35 kU_A_/l) sIgE:s divided by the number of positive tests among IgE-sensitized individuals. For evaluation of differences in median levels of IgE to specific allergens between females and males, quantile regression was used. Generalized estimating equations (GEEs) [27] with an unstructured correlation matrix were used to assess the impact of sex over time for IgE-sensitization to foods and airborne allergens. Potential confounders [28–31] were tested using exploratory backward stepwise logistic regression. None of the tested factors; parental allergy, low birthweight (< 2500 g), exclusive breast-feeding ≥ 4 months, maternal smoking during pregnancy and at enrollment, low socioeconomic status, young mother (< 26 years) and atopic dermatitis before age 4 years confounded the association between sex and IgE-sensitization. All statistical analyses were performed with STATA Statistical Software (release 14.2; Stata-Corp, College Station, TX, USA).

## Results

### IgE-sensitization at age 24 years

**Fig. 2:**
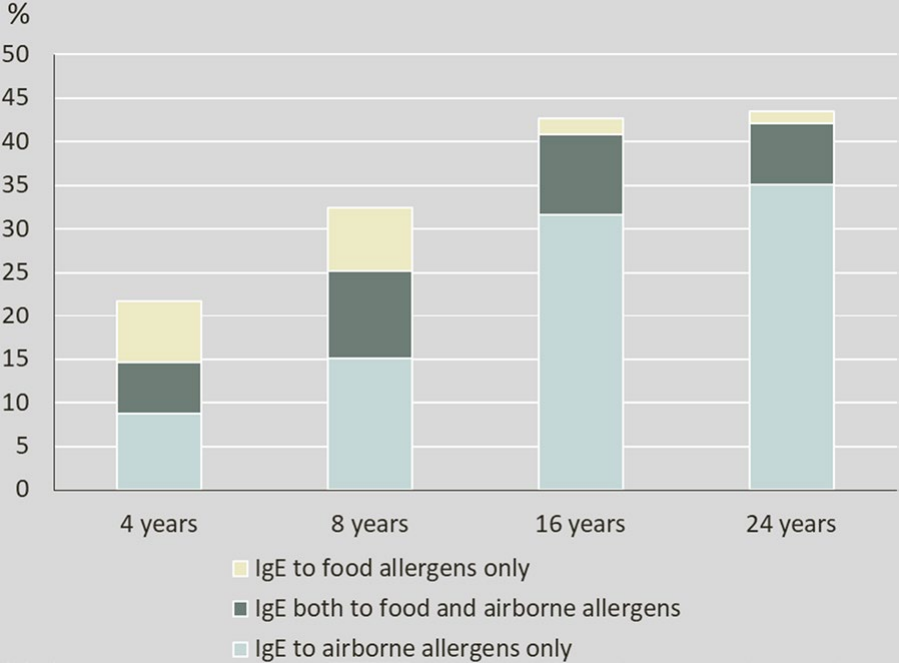
Prevalence of IgE-sensitization to food* and airborne** allergens at ages 4, 8, 16 and 24 years in the population-based cohort BAMSE. *Peanut, soy, wheat, milk, egg and cod. **Timothy, birch, cat, dog, house dust mites, mugwort, horse and *Cladosporium herbarum*

**Fig. 3:**
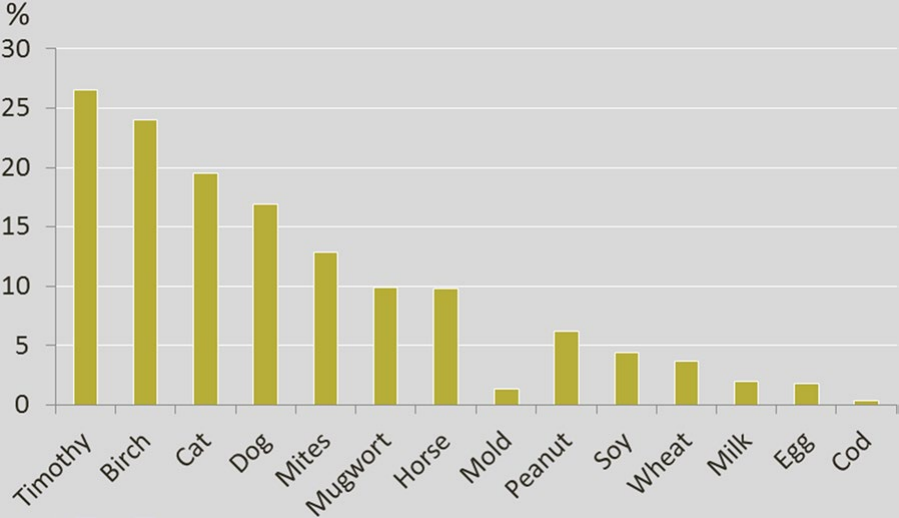
Proportions of individuals with sIgE-sensitization at the 24-year follow-up in the population-based birth cohort BAMSE (n = 2234). Timothy n = 594, birch n = 541, cat n = 438, dog n = 379, mites n = 290, mugwort n = 221, horse n = 219, mold n = 30, peanut n = 138, soy n = 98, wheat n = 82, milk n = 44, egg n = 42, cod n = 7

At age 24 years 43.4% (970/2234) were sensitized to any of the tested foods or airborne allergens. This proportion was in the same range as at the 16-year follow-up, and higher compared with ages 4 and 8 years, Fig. 2. Sensitization to foods had decreased compared to previous follow-ups affecting 8.4%, while sensitization to airborne allergens was more common at the 24-year follow-up, affecting 42.2%. Thirty-five percent were sensitized to airborne allergens only, 1.3% to foods only and 7.1% both to foods and airborne allergens. Timothy and birch were the most prevalent sensitizing allergens (26.6% and 24.2%, respectively), Fig. 3. The median level of sIgE for foods was 2.5 kU_A_/l (25th percentile 1.2 kU_A_/l, 75th percentile 13.5 kU_A_/l) and for airborne allergens 6.4 kU_A_/l (25th percentile 2.4 kU_A_/l, 75th percentile 14.7 kU_A_/l).

### Longitudinal analyses—impact of sex on IgE-sensitization over time, up to age 24 years

**Fig. 4:**
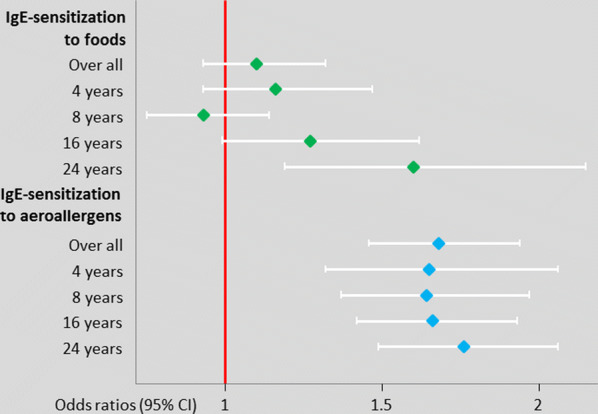
Impact of male sex on IgE-sensitization up to age 24 years in the BAMSE cohort. GEE analyses include individuals with complete data on IgE-sensitization from at least two follow-ups, 1430 males and 1473 females (reference group)

We evaluated the association between sex and IgE-sensitization over time using generalized estimating equations (GEE) with females as the reference category and results are shown in Fig. 4. Sex was not significantly associated with sensitization to foods up to 16 years. However, at 24 years significantly more males than females had IgE-sensitization to foods. Evaluation of the whole time period revealed no statistically significant association between sex and IgE-sensitization to foods (over all OR: 1.10, 95% CI 0.93–1.32), Fig. 4. In contrast, male sex was significantly associated with IgE-sensitization to airborne allergens at all ages (over all OR: 1.68, 95% CI 1.46–1.94).

### IgE-sensitization to specific allergens at 4, 8, 16 and 24 years in relation to sex

**Fig. 5:**
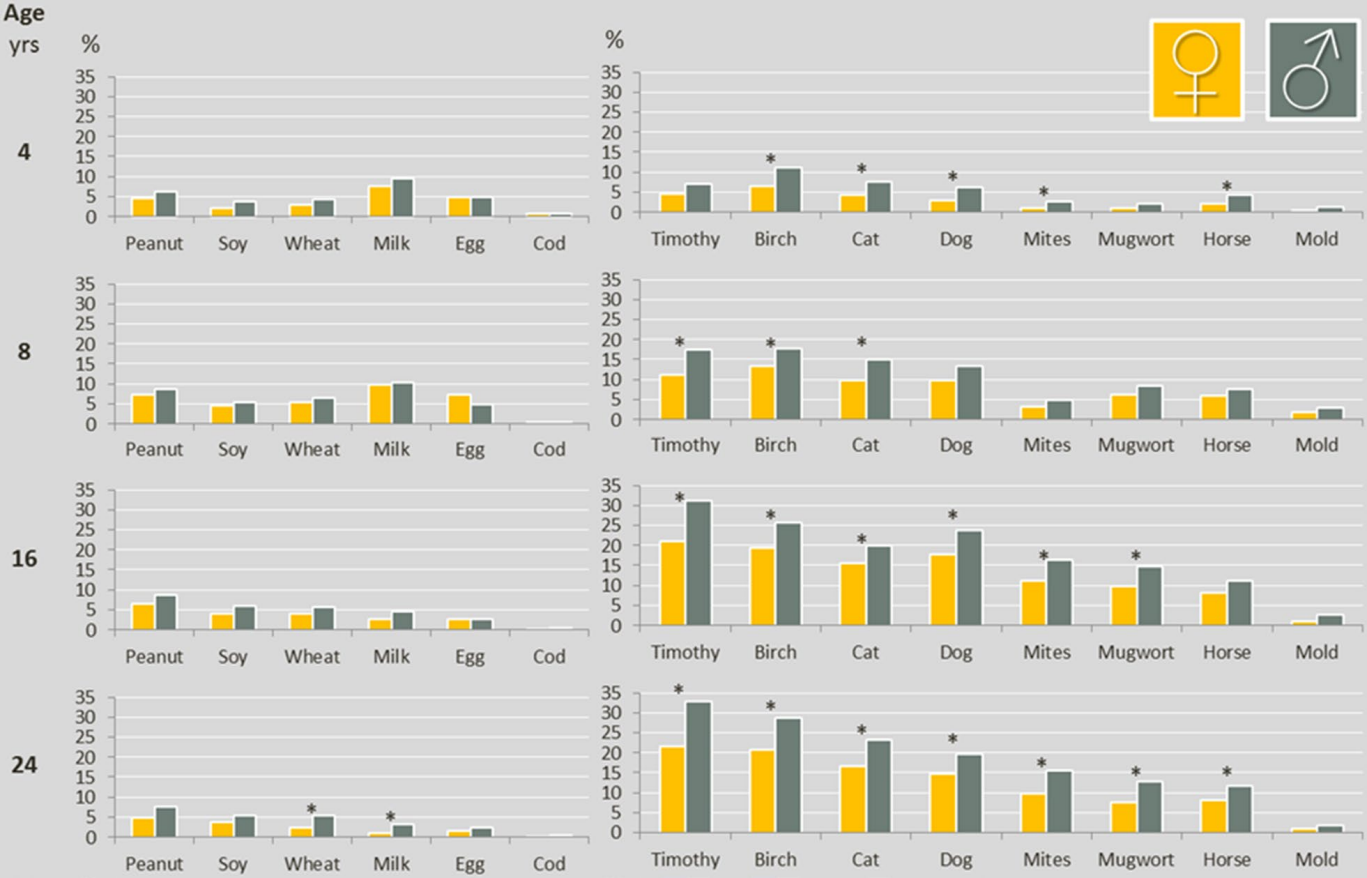
Proportions of females and males with sensitization (IgE ≥ 0.35 kU_A_/l) to specific foods and airborne allergens at ages 4 years (n = 2604), 8 years (n = 2446), 16 years (n = 2547) and 24 years (n = 2234), in the BAMSE birth cohort. * Statistical significant difference, based on Bonferroni correction (p ≤ 0.004)

The prevalences of IgE-sensitization to specific food allergens among females and males at ages 4, 8, 16 and 24 years are shown in Fig. 5. Significantly more males were sensitized to milk and wheat at age 24 years. However, there were no significant differences between females and males regarding IgE-sensitizations to specific foods at ages 4 years, 8 years and 16 years using a stringent Bonferroni correction cut-off (p ≤ 0.004). With respect to airborne allergens, more males than females were sensitized and for many airborne allergens there were significant differences (p ≤ 0.004) between females and males already at age 4 years. At age 24 years significantly more males than females were sensitized to timothy, birch, cat, dog, mugwort, mites and horse (all p < 0.004). Comparison of sIgE-levels for the 14 allergens between females and males at age 24 years showed no significant differences, (all p > 0.004), Additional file 1: Table S1. The highest level of sIgE was against birch for both females (median level 6.5 kU_A_/l (25th percentile 1.5 kU_A_/l, 75th percentile 28.0 kU_A_/l) and males (median level 13.0 kU_A_/l (25th percentile 2.3 kU_A_/l, 75th percentile 40.0 kU_A_/l), and tended to be higher among males, p = 0.005. Sex was not significantly associated with sIgE-levels at ages 4, 8 and 16 years, all p > 0.004, data not shown.

### Sensitivity analysis

At the 24-year follow-up more females (n = 1244, 61%) than males (n = 990, 48%) attended the clinical examination and provided blood for analysis of sIgE. In view of this sex-related difference in response rates we performed a sensitivity analysis and compared the prevalence of IgE-sensitization, atopic dermatitis, asthma and rhinitis [5] at previous follow-ups for females and males who attended the clinical examination at age 24 years with the original cohort, Additional file 1: Table S2. Overall, differences were minor for both females and males, although atopic dermatitis, rhinitis and IgE-sensitization were somewhat more common, especially at recent follow-ups, among both females and males who attended the clinical examination at the 24-year follow-up. However, differences were not more pronounced among males.

We also explored potential differences between males and females regarding some environmental exposures early in life. In the study population, 83% attended kindergarten at the 2-year follow up. Differences between boys and girls were only minor (p = 0.19). Further, we found that a similar proportion of girls and boys had a cat or dog at home both at inclusion (2 months), at age 1 year, 2 years and 4 years (all p-values non-significant).

## Discussion

This Swedish longitudinal population-based study shows that almost one in two young adults (43.4%) is sensitized to at least one specific allergen. Sensitization to airborne allergens continues to increase with age up to young adulthood, whereas sensitization to food allergens seems to level off. Sensitization to airborne allergens was significantly more prevalent among males than females from early childhood up to adulthood, while there were only minor differences for food allergens.

The prevalence rates of IgE-sensitization over time in our study are similar to European studies with a follow-up time of 18 and 19 years [7, 8]. However, our study goes beyond childhood and adolescence and we show that the overall prevalence of IgE-sensitization remained rather unchanged from late adolescence up to age 24 years. A new finding of our study is that even though male sex was strongly associated with IgE-sensitization to airborne allergens at all ages, there was little evidence for significant associations between sex and sensitization to foods. Most large population-based longitudinal studies included IgE-sensitization to airborne allergens [7–9, 14, 32] while few included both foods and airborne allergens [8, 9, 16]. Moreover, when evaluation of differences in IgE-sensitization between females and males was done, sensitization to foods and airborne allergens were often analyzed together. Salo et al. [4] explored IgE-sensitization to foods in relation to sex among 1–5 years old children in a large population-based cross-sectional study from the US and found no significant difference, which is in line with our results. However, in the same study male sex in individuals 6 years or older was significantly associated with IgE-sensitization to one or more foods, in particular shrimp and peanut [4]. The authors also noted that IgE-sensitization to house dust mites and birch was significantly more common among males 6 years or older. Part of the association between IgE-sensitization to shrimp and peanut among males is probably explained by serological IgE cross-reactivity with house dust mites and birch, respectively [33, 34]. Similarly, some of the sex differences regarding wheat sensitization at age 24 years found in our study could likely be explained by serological IgE cross-reactivity with timothy [35]. The discrepancy regarding the associations between sex and IgE-sensitization to foods and airborne allergens we found is intriguing and can probably not be explained by general differences in allergen exposure, although we know that the intake of some dietary components, such as antioxidants, may differ between males and females [36]. The observed sex-differences in IgE sensitization to milk at 24 years is difficult to interpret due to low numbers of affected individuals.

We also explored whether sIgE levels differed between males and females, but found no significant differences although sIgE to birch was higher in males (median 13.0 kU_A_/L vs 6.5 kU_A_/L, borderline significant after multiple test correction). Our findings are similar to results from Salo et al. [4] (N = 7268) who compared sIgE levels for 19 sIgEs in relation to sex among participants aged 6 years or older. Only two sIgEs (milk and *Aspergillus fumigatus*) differed by sex (both p < 0.05 and > 0.001).

Genetic factors are known to be strongly associated with the risk of developing sensitization in children [37]. Also, epigenetic changes are linked to atopy and high total IgE levels [38]. However, it is currently not known if sensitization to foods truly has different genetic background than that to aeroallergens, since most identified loci for sensitization overlap with those identified for other allergy-related diseases. Furthermore, in the larger GWAS studies to date, sensitization to foods and aeroallergens has been analyzed jointly [37, 39]. Sex-specific genetic effects on sensitization and allergic diseases have been reported in the literature [40–42] but no consistent picture or explanation of the underlying biology has emerged. Whether there are primarily genetic, hormonal or environmental factors associated with the observed sex differences in IgE-sensitization in our study remain to be further investigated.

Strengths of our study include the population-based design, long follow-up time, limited loss to follow-up and that blood was collected for analyses of sIgE including both food and airborne allergens at four time points. We applied a stringent multiple-test correction approach in order to identify robust differences in IgE-sensitization between males and females. We evaluated 14 allergens covering the most common food and airborne allergens to ensure that most IgE-sensitized individuals were detected. However, some individuals with less common IgE-sensitizations could have been miss-classified as non-sensitized in our study. Similar to most longitudinal cohort studies, potential selection bias needs to be taken into account in our study, especially since more males than females were lost to follow up at the recent 24-year follow up. We therefore evaluated whether selection bias differed between females and males. The willingness to participate was somewhat higher among individuals with IgE-sensitization, atopic dermatitis and rhinitis especially at older ages. However, difference between females and males were minor. Thus, selection bias is unlikely to explain the sex differences found in our study.

In summary, we report that IgE-sensitization to airborne allergens continues to increase with age up to young adulthood, whereas sensitization to food allergens seems to level off. At all ages, sensitization to airborne allergens was more common in males compared to females. Further analyses of the underlying determinants for the differences in IgE sensitization between females and males are warranted.

## Supplementary information

**Additional file 1: Table S1.** IgE-levels (percentiles) among females and males with sensitization (IgE ≥ 0.35 kU_A_/l) to specific food and airborne allergens at 24 years (n = 2234), in the BAMSE birth cohort. Statistical difference calculated by use of quantile regression and a p-value lower than 0.004 was considered significant based on Bonferroni correction (p ≤ 0.004). **Table S2.** Comparison of females and males in the original cohort and the study population that provided blood at the 24 year follow-up

## Abbreviations

AD: Atopic dermatitis
BAMSE: Barn/Children, Allergy, Milieu, Stockholm, Epidemiology
sIgE: Allergen specific immunoglobulin E antibodies
