# Supplementary material for: Male sex is strongly associated with IgE-sensitization to airborne but not food allergens: results up to age 24 years from the BAMSE birth cohort
Source: Clin Transl Allergy. 2020 May 25;10:15. doi: 10.1186/s13601-020-00319-w (PMC7247167; doi:10.1186/s13601-020-00319-w)
Supplement: Supplementary file 1 — Additional file 1: Table S1. IgE-levels (percentiles) among females and males with sensitization (IgE ≥ 0.35 kUA/l) to specific food and airborne allergens at 24 years (n = 2234), in the BAMSE birth cohort. Statistical difference calculated by use of quantile regression and a p-value lower than 0.004 was considered significant based on Bonferroni correction (p ≤ 0.004). Table S2. Comparison of females and males in the original cohort and the study population that provided blood at the 24 year follow-up [file 13601_2020_319_MOESM1_ESM.docx]

**Table S1**. IgE-levels (percentiles) among females and males with sensitization (IgE ≥0.35 kU_A_/l) to specific food and airborne allergens at 24 years (n=2,234), in the BAMSE birth cohort. Statistical difference calculated by use of quantile regression and a p-value lower than 0.004 was considered significant based on Bonferroni correction (p=<0.004).

|  |  | Females |  |  |  |  | Males |  |  |  |  |  |
| --- | --- | --- | --- | --- | --- | --- | --- | --- | --- | --- | --- | --- |
|  |  | **n** | **IgE-levels, kU_A_/l, percentiles** | | |  | **n** | **IgE-levels, kU_A_/l, percentiles** | | |  | **P-value** |
| Allergen |  |  | median | 25:th | 75:th |  |  | median | 25:th | 75:th |  |  |
| Timothy |  | 268 | 4.4 | 1.2 | 19.0 |  | 326 | 5.9 | 1.5 | 23.0 |  | 0.27 |
| Birch |  | 258 | 6.5 | 1.5 | 28.0 |  | 283 | 13.0 | 2.3 | 40.0 |  | 0.005 |
| Cat |  | 208 | 4.3 | 1.4 | 10.5 |  | 230 | 4.2 | 1.6 | 12.0 |  | 0.89 |
| Dog |  | 184 | 1.4 | 0.65 | 4.0 |  | 195 | 1.5 | 0.67 | 3.3 |  | 0.68 |
| Mites |  | 130 | 0.93 | 0.66 | 2.4 |  | 160 | 1.4 | 0.71 | 6.5 |  | 0.14 |
| Mugwort |  | 95 | 1.1 | 0.57 | 2.5 |  | 126 | 0.93 | 0.53 | 2.8 |  | 0.41 |
| Horse |  | 102 | 2.0 | 0.83 | 5.6 |  | 117 | 3.4 | 0.91 | 7.0 |  | 0.04 |
| Mold |  | 13 | 1.4 | 0.96 | 8.6 |  | 17 | 0.91 | 0.67 | 1.3 |  | 0.70 |
| Peanut |  | 62 | 6.05 | 1.4 | 57.0 |  | 76 | 6.4 | 1.5 | 19.5 |  | 0.91 |
| Soy |  | 45 | 0.82 | 0.62 | 2.4 |  | 53 | 1.2 | 0.57 | 1.8 |  | 0.21 |
| Wheat |  | 29 | 0.79 | 0.49 | 1.5 |  | 53 | 1.0 | 1.55 | 2.6 |  | 0.59 |
| Milk |  | 12 | 0.68 | 0.49 | 2.05 |  | 32 | 1.05 | 0.64 | 2.2 |  | 0.61 |
| Egg |  | 19 | 0.52 | 0.43 | 1.0 |  | 23 | 0.5 | 0.5 | 1.4 |  | 0.32 |
| Cod |  | 2 | 10.2 | 0.35 | 20.0 |  | 5 | 0.78 | 0.47 | 6.3 |  | na |

**Table S2.** Comparison of females and males in the original cohort and the study population that provided blood at the 24 year follow-up

|  |  | Females | | | | | | | |  | Males | | | | | | | |
| --- | --- | --- | --- | --- | --- | --- | --- | --- | --- | --- | --- | --- | --- | --- | --- | --- | --- | --- |
|  |  | **Original cohort**  **n=2,024** | |  | **Study population**  **IgE 24 yrs**  **n=1,244** | | | | |  | **Original cohort**  **n=2,065** | |  | **Study population**  **IgE 24 yrs**  **n=990** | | | | |
|  |  | OBS | % |  | OBS | % | 95% |  | CI |  | OBS | % |  | OBS | % | 95% |  | CI |
| AD^†^ 4 yrs |  | 1,842 | 20.8 |  | 1,181 | 21.3 | 19.0 | - | 23.7 |  | 1,879 | 20.5 |  | 944 | 21.0 | 18.4 | - | 23.6 |
| AD^†^ 8 yrs |  | 1,643 | 17.3 |  | 1,083 | 17.3 | 15.0 | - | 19.5 |  | 1,687 | 15.4 |  | 878 | 15.0 | 12.7 | - | 17.4 |
| AD^†^ 16 yrs |  | 1,592 | 9.7 |  | 1,098 | 11.4 | 9.5 | - | 13.3 |  | 1,572 | 6.7 |  | 880 | 7.6 | 5.9 | - | 9.4 |
|  |  |  |  |  |  |  |  |  |  |  |  |  |  |  |  |  |  |  |
| Asthma 4 yrs |  | 1,836 | 5.3 |  | 1,180 | 4.9 | 3.7 | - | 6.2 |  | 1,868 | 8.7 |  | 941 | 8.7 | 6.9 | - | 10.5 |
| Asthma 8 yrs |  | 1,683 | 5.4 |  | 1,108 | 5.5 | 4.2 | - | 6.9 |  | 1,714 | 7.2 |  | 891 | 7.2 | 5.5 | - | 8.9 |
| Asthma 16 yrs |  | 1,573 | 6.9 |  | 1,085 | 7.3 | 5.7 | - | 8.8 |  | 1,542 | 5.9 |  | 864 | 6.0 | 4.4 | - | 7.6 |
|  |  |  |  |  |  |  |  |  |  |  |  |  |  |  |  |  |  |  |
| Rhinitis 4 yrs |  | 1,825 | 8.6 |  | 1,166 | 9.2 | 7.5 | - | 10.8 |  | 1,857 | 12.3 |  | 934 | 12.3 | 10.2 | - | 14.4 |
| Rhinitis 8 yrs |  | 1,680 | 11.3 |  | 1,105 | 12.4 | 10.5 | - | 14.3 |  | 1,714 | 15.4 |  | 891 | 15.8 | 13.4 | - | 18.2 |
| Rhinitis 16 yrs |  | 1,563 | 22.6 |  | 1,075 | 24.6 | 22.0 | - | 27.1 |  | 1,546 | 28.2 |  | 867 | 29.6 | 26.6 | - | 32.7 |
|  |  |  |  |  |  |  |  |  |  |  |  |  |  |  |  |  |  |  |
| Any IgE-sens 4 yrs |  | 1,281 | 19.4 |  | 878 | 20.8 | 17.2 | - | 23.5 |  | 1,323 | 24.2 |  | 732 | 24.6 | 21.9 | - | 27.7 |
| Any IgE-sens 8 yrs |  | 1,191 | 29.9 |  | 847 | 31.8 | 27.3 | - | 34.9 |  | 1,255 | 34.8 |  | 743 | 35.4 | 32.2 | - | 38.8 |
| Any IgE-sens 16 yrs |  | 1,311 | 36.8 |  | 1,001 | 37.9 | 34.2 | - | 40.9 |  | 1,236 | 48.9 |  | 778 | 49.1 | 46.1 | - | 52.6 |

^†^Atopic dermatitis
